# *FLT3*^*N676K*^ drives acute myeloid leukemia in a xenograft model of *KMT2A-MLLT3* leukemogenesis

**DOI:** 10.1038/s41375-019-0465-1

**Published:** 2019-04-05

**Authors:** Axel Hyrenius-Wittsten, Mattias Pilheden, Antoni Falqués-Costa, Mia Eriksson, Helena Sturesson, Pauline Schneider, Priscilla Wander, Cristian Garcia-Ruiz, Jian Liu, Helena Ågerstam, Anne Hultquist, Henrik Lilljebjörn, Ronald W. Stam, Marcus Järås, Anna K. Hagström-Andersson

**Affiliations:** 1 Division of Clinical Genetics, Department of Laboratory Medicine, Lund University, Lund, Sweden; 2 Princess Máxima Center for Pediatric Oncology, Utrecht, The Netherlands; 3 Department of Pathology, Skane University Hospital, Lund University, Lund, Sweden; 4 Lund Stem Cell Center, Lund University, Lund, Sweden

**Keywords:** Cancer models, Leukaemia

## To the Editor:

Activating signaling mutations are common in acute leukemia with *KMT2A* (previously *MLL*) rearrangements (*KMT2A-*R) [1]. When defining the genetic landscape of infant *KMT2A*-R acute lymphoblastic leukemia (ALL), we identified a novel *FLT3*^*N676K*^ mutation in both infant ALL and non-infant acute myeloid leukemia (AML) [1]. *FLT3*^*N676K*^ was the most common *FLT3* mutation in our cohort and we recently showed that it cooperates with *KMT2A-MLLT3* in a syngeneic mouse model [1, 2]. To study the ability of *FLT3*^*N676K*^ to cooperate with *KMT2A-MLLT3* in human leukemogenesis, we transduced human CD34^+^-enriched cord blood (CB) cells and followed leukemia development immunophenotypically and molecularly in NOD.Cg-*Prkdc*^*scid*^*Il2rg*^*tm1Wjl*^/SzJ (NSG) mice.

**Fig. 1:**
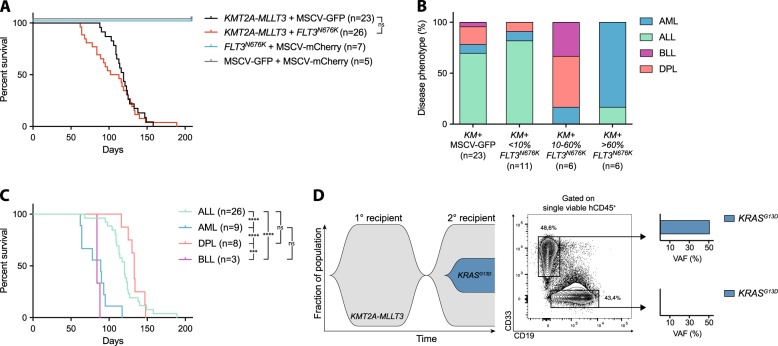
*FLT3*^*N676K*^ alters the lineage distribution of *KMT2A-MLLT3-*driven leukemia. **a** Kaplan–Meier survival curves of NSG mice transplanted with CD34^+^ cord blood cells cotransduced with *KMT2A-MLLT3* *+* *FLT3*^*N676K*^ (*n* = 23), *KMT2A-MLLT3* *+* MSCV-GFP (*n* = 26, of which three died and no tissue samples could be collected), *FLT3*^*N676K*^ *+* MSCV-mCherry (*n* = 7), or MSCV-GFP *+* MSCV-mCherry (*n* = 5). **b** Distribution of mice that succumbed to ALL, AML, DPL, or BLL within *KMT2A-MLLT3* *+* MSCV-GFP and *KMT2A-MLLT3*-mCherry *+* *FLT3*^*N676K*^-GFP recipients divided on the fraction of mCherry^+^GFP^+^ (<10%, *n* = 11; 10–60%, *n* = 6; or > 60%, *n* = 6) cells within hCD45^+^ bone marrow (BM) cells. **c** Kaplan–Meier survival curves of all xenograft leukemias based on their immunophenotype showed an accelerated disease for AML as compared with both ALL (*P* *<* 0.0001) and DPL (*P* *<* 0.0001). **d** Fish plot showing progression of one *KMT2A-MLLT3* + MSCV-GFP BLL that gained a *de novo* *KRAS*^*G13D*^ (VAF 17% in hCD45^+^ BM cells) in the secondary recipient (h11.13-1) and targeted resequencing of hCD45^+^CD19^−^CD33^+^ and hCD45^+^CD19^+^CD33^−^ BM cells from the secondary recipient (h11.13-1) revealed the *KRAS*^*G13D*^ mutation to reside in CD19^−^CD33^+^ leukemia cells (VAF 51%). Mantel–Cox log-rank test, ***P ≤ 0.001, ****P ≤ 0.0001, ns = not significant

Mice that received *KMT2A-MLLT3* with or without *FLT3*^*N676K*^ developed a lethal leukemia, often with splenomegaly, thrombocytopenia, and leukocytosis, with no difference in median disease latency (107.5 and 119 days, respectively, *P* = 0.48) and mice receiving *FLT3*^*N676K*^ alone showed no sign of disease (Fig. 1a, Supplementary Fig. 1A–E, and Supplementary Data 1). Leukemic mice succumbed to ALL (> 50% CD19^+^CD33^−^), AML (> 50% CD19^−^CD33^+^), double-positive leukemia (DPL, > 20% CD19^+^CD33^+^), or bilineal leukemia (BLL, <50% CD19^+^CD33^−^, < 50% CD19^−^CD33^+^, and < 20% CD19^+^CD33^+^); thus, the leukemias often coexisted with leukemia cells of another immunophenotype (Supplementary Fig. 1F, G and Supplementary Data 1) [3–5]. Previous studies have shown that retroviral overexpression of *KMT2A-MLLT3* in human CB cells in NOD.CB17/*Prkdc*^*scid*^ (NOD/SCID), NOD.Cg-*Prkdc*^*scid*^*B2m*^*tm1Unc*^ (NOD/SCID-B2m), or NSG immunodeficient mice, primarily gives rise to ALL, sometimes to leukemias expressing both lymphoid and myeloid markers or bilineal leukemias, but rarely AML [3–6]. *KMT2A-MLLT3-*driven AML can only be generated with high penetrance, and re-transplanted in immunodeficient mice transgenically expressing human myeloid cytokines, consistent with the idea that external factors can influence the phenotype of the developing leukemia [3, 6]. In agreement, most recipients that received *KMT2A-MLLT3* alone developed ALL (16/23, 69.6%) or DPL (4/23, 17.4%) and AML was rare (2/23, 8.7%) [3, 4, 6]. By contrast, five out of six recipients that received *KMT2A-MLLT3+FLT3*^*N676K*^ and that had > 60% (*n* = 6, range 60.4–94.1%) of co-expressing cells, developed AML and one developed ALL (Fig. 1b, Supplementary Fig. 1H, I, and Supplementary Data 1). Thus, *FLT3*^*N676K*^ preferentially drives myeloid expansion, similar to mutant *Flt3* in a syngeneic setting [7]. FLT3 is expressed in human hematopoietic stem and progenitor cells, with the highest expression in granulocyte–macrophage progenitors (GMPs), and its signaling supports survival of those cells [8]. Combined, this suggests that *FLT3*^*N676K*^ affects the survival of myeloid progenitors. In this context, it is interesting to note that FLT3 tyrosine kinase domain mutations are enriched in pediatric AML with *KMT2A-MLLT3* [9, 10]. Most recipients with < 10% (*n* = 11, range 0.2–5.3%) of co-expressing *KMT2A-MLLT3* + *FLT3*^*N676K*^ cells succumbed to ALL, consistent with leukemia being driven by *KMT2A-MLLT3* alone. Among those with 10–60% (*n* = 6, range 10.9–31.6%) of co-expressing cells, a mixture of diseases developed since leukemia could be driven both by *KMT2A-MLLT3* alone and *KMT2A-MLLT3* *+* *FLT3*^*N676K*^ (Fig. 1b, Supplementary Fig. 1H, I, and Supplementary Data 1).

Co-expression of *KMT2A-MLLT3* and *FLT3*^*N676K*^ preferentially expanded myeloid cells (*P* < 0.0001, Supplementary Fig. 1J) and a high proportion of CD19^−^CD33^+^ cells at sacrifice, across the cohort, correlated with accelerated disease (*r*_s_ = −0.6537, *P* < 0.0001, Supplementary Fig. 1K, L). In agreement, AML developed with significantly shorter latency as compared with ALL and DPL (89 vs. 120 and 133 days, respectively, both *P* < 0.0001), but not with BLL (84 days, Fig. 1c). Further, *FLT3*^*N676K*^-driven AML had a tendency toward shorter survival (median latency 78 days, range 62–117 days vs. 93 days for *KMT2A-MLLT3* alone, Supplementary Data 1).

To determine the evolution of phenotypically distinct leukemia cells in secondary recipients, BM cells from six primary *KMT2A-MLLT3* *+* *FLT3*^*N676K*^ leukemias (three each with > 60% or 20–32% of *FLT3*^*N676K*^-expressing cells) and from four primary *KMT2A-MLLT3* leukemias, were re-transplanted. All leukemias gave rise to secondary malignancies and recipients that received BM from AML (*n* = 2) and ALL (*n* = 1) with > 60% of *KMT2A-MLLT3* *+* *FLT3*^*N676K*^ cells had an accelerated disease onset and maintained leukemia immunophenotype (median latency of 117 and 41 days, for the primary and secondary recipients, respectively) (Supplementary Fig. 2A-E and Supplementary Data 2). Thus, *FLT3*^*N676K*^ circumvented the cytokine dependence normally required for myeloid cells in immunodeficient mice [3]. By contrast, all secondary recipients that received BM with 20–32% *KMT2A-MLLT3* *+* *FLT3*^*N676K*^ cells succumbed to ALL, irrespective of disease phenotype in the primary recipients (one AML and two BLL). Thus, the myeloid *FLT3*^*N676K*^-expressing cells unexpectedly decreased in size, while the *KMT2A-MLLT3-*expressing lymphoid cells increased to clonal dominance (Supplementary Fig. 2F, G and Supplementary Data 2). This suggests that the *FLT3*^*N676K*^*-*containing myeloid leukemia population needs to be sufficiently large to expand in secondary recipients, either because they otherwise are outcompeted by the larger population of more easily engrafted ALL cells, or because they themselves need to mediate the permissive microenvironment that allows myeloid cells to engraft. Similarly, in all but one of the secondary recipients that received BM from leukemias expressing only *KMT2A-MLLT3* (two AML, one ALL, and one BLL), the disease phenotype changed and myeloid cells did not engraft (Supplementary Fig. 2H-J and Supplementary Data 2).

In the secondary recipient with maintained immunophenotype (a BLL, h11.13-1), the myeloid cells unexpectedly expanded from 38% to close to 50% (Fig. 1d and Supplementary Data 2). This suggested that the myeloid cells had acquired a de novo mutation that allowed serial transplantation, similar to what was observed for *FLT3*^*N676K*^. Strikingly, targeted sequencing of AML-associated genes on hCD45^+^ BM from this mouse identified a *KRAS*^*G13D*^ in 34% of the cells. Resequencing of hCD45^+^CD19^−^CD33^+^ and hCD45^+^CD19^+^CD33^−^ BM showed that *KRAS*^*G13D*^ was present exclusively in the myeloid population and based on the variant allele frequency of 51%, that all cells carried the mutation (Fig. 1d and Supplementary Table 1, 2). Further, *KRAS*^*G13D*^ likely arose independently in h11.13-1 as no mutation was identified, at the level of our detection, in the primary (h11.13) or in a separate secondary recipient (h11.13-2) from the same primary mouse (h11.13) that developed ALL (Supplementary Table 2 and Supplementary Data 2).

**Fig. 2:**
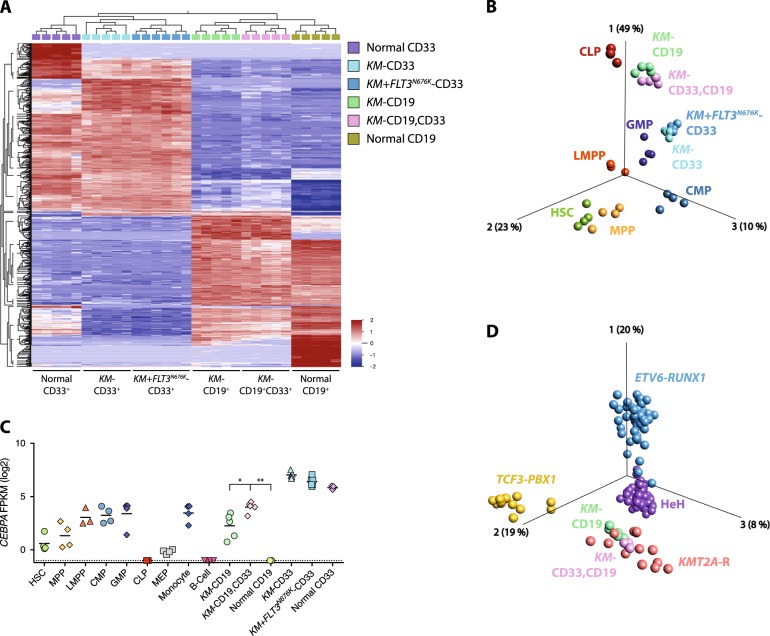
DPL cells are ALL cells with aberrant CD33 expression. **a** Hierarchical clustering based on multigroup comparison of myeloid CD19^−^CD33^+^ leukemia cells from *KMT2A-MLLT3* *+* MSCV-GFP (*KM-*CD33) and *KMT2A-MLLT3* *+* *FLT3*^*N676K*^ (*KM* + *FLT3*^*N676K*^*-*CD33), lymphoid CD19^+^CD33^−^ leukemia cells from *KMT2A-MLLT3* *+* MSCV-GFP (*KM-*CD19), and double-positive CD19^+^CD33^+^ leukemia cells from *KMT2A-MLLT3* *+* MSCV-GFP (*KM-*CD19,CD33), as well as normal myeloid- CD19^−^CD33^+^ (normal CD33) and lymphoid CD19^+^CD33^−^ (normal CD19) cells from MSCV-GFP *+* MSCV-mCherry using 637 variables (*P* = 2.2e^−15^, FDR = 4.9e^−14^). **b** Supervised (1500 variables, *P* = 3.6e^−4^, FDR = 3.1e^−3^) PCA based on human hematopoietic stem cells (HSC), multipotent progenitors (MPP), lymphoid-primed multipotent progenitors (LMPP), common myeloid progenitors (CMP), granulocyte–macrophage progenitors (GMP), and common lymphoid progenitors (CLP) [11]. Samples with leukemia cells from *KM-*CD33, *KM* + *FLT3*^*N676K*^*-*CD33, *KM-*CD19, and *KM-*CD19,CD33 were inserted into the same PCA (still based solely on the normal populations [11]), revealing that AML cells mainly resembled GMPs and that both ALL and DPL mainly resembled CLPs. **c** *CEBPA* expression (FPKM log2) within sorted leukemia, normal populations, and within HSC, MPP, LMPP, CMP, GMP, CLP, MEP, monocytes, and B cells [11]. **d** Supervised (1501 variables, *P* = 3.3e^−10^, FDR = 3.1e^−9^) PCA based on pediatric BCP-ALL with *ETV6-RUNX1*, high hyperdiploid (HeH), *TCF3-PBX1*, or *KMT2A-*R [12]. Samples with leukemia cells from *KM-*CD19 and *KM-*CD19,CD33 were inserted into the same PCA (still based solely on the pediatric BCP-ALL populations [12]). Mann–Whitney U test used in (**c**), *P ≤ 0.05, **P ≤ 0.01, ns = not significant

Gene expression profiling (GEP) followed by principal component analysis (PCA) showed that the leukemias segregated based on their immunophenotype, with an evident separation between leukemias and normal hematopoietic cells (Fig. 2a, Supplementary Fig. 3A, B, and Supplementary Table 3). All leukemias expressed high levels of known *KMT2A*-R target genes and showed enrichment of gene signatures associated with primary *KMT2A*-R leukemia, indicating that they maintain a GEP representative of human disease (Supplementary Fig. 3C, D and Supplementary Data 3–6). In line with the hypothesis that *KMT2A-MLLT3* DPL cells are ALL cells with aberrant CD33 expression, they clustered closely with ALL cells. Both populations expressed high levels of ALL-associated cell surface markers and lymphoid transcription factors (Fig. 2a and Supplementary Fig. 3B, E-H). Further, *CD33* and other AML-associated cell surface markers and key myeloid transcription factors, all showed lower expression in DPL cells as compared with normal myeloid- and AML cells (Supplementary Fig. 3G-I).

By correlating the GEPs of the xenograft leukemias to those of normal hematopoietic cells [11], DPL and ALL were found to resemble normal common lymphoid progenitors (CLPs) and AML cells normal GMPs (Fig. 2b). Further, ALL and DPL cells had significantly higher expression of the transcription factor *CEBPA* as compared with normal lymphoid cells which lacked *CEBPA* expression (Fig. 2c). *CEBPA* drives myeloid programs and is expressed in most hematopoietic progenitors, in particular in GMPs, with CLPs lacking *CEBPA* expression (Fig. 2c) [11]. Finally, to link the GEPs of our xenograft leukemias to those of pediatric *KMT2A*-R leukemia, we utilized a dataset of pediatric B-cell precursor ALL (BCP-ALL) [12]. Multigroup comparison visualized by PCA showed that *KMT2A-MLLT3* DPL and ALL mainly resembled *KMT2A*-R BCP-ALL (Fig. 2d), again highlighting that the xenograft leukemias resemble human leukemia.

We next studied the transcriptional changes induced by *FLT3*^*N676K*^ in *KMT2A-MLLT3* AML cells. Gene set enrichment analysis (GSEA) revealed enrichment of gene sets connected to the Myc-transcriptional network, cell cycle, and proliferation when compared with *KMT2A-MLLT3* AML (Supplementary Data 7). Similar to our previous findings in a syngeneic *KMT2A-MLLT3* mouse model [2], the Myc-centered program [13] was not linked to the pluripotency network (Supplementary Fig. 4A and Supplementary Data 8, 9). Since FLT3 mutations activate mitogen-activated protein kinase (MAPK) signaling [14], we investigated if *FLT3*^*N676K*^ increased expression of MEK/ERK-pathway genes, by studying the expression of known transcriptional output genes and negative feedback regulators of the pathway [15]. Indeed, enrichment of MEK/ERK-associated genes was evident in *FLT3*^*N676K*^-expressing cells, suggesting that *FLT3*^*N676K*^ allows cells to overcome normal feedback regulation, leading to sustained signaling [15] (Supplementary Fig. 4B). Finally, *FLT3*^*N676K*^-expressing AML showed preserved transcriptional changes to those seen in infant *KMT2A*-*AFF1* ALL with activating mutations [1] (Supplementary Fig. 4C). Thus, *FLT3*^*N676K*^ may circumvent the cytokine dependence seen for myeloid cells in immunodeficient mice by providing constitutive active signaling promoting cell proliferation, likely through the MAPK/ERK pathway.

Herein, we demonstrate that co-expression of *KMT2A-MLLT3* and *FLT3*^*N676K*^ in human CB cells primarily causes AML and thus alters the lineage distribution of *KMT2A-MLLT3-*driven leukemia. AML could only be serially transplanted with maintained immunophenotype in the presence of *FLT3*^*N676K*^. This is consistent with the idea that activated signaling allows myeloid cells to more efficiently engraft and maintain their self-renewal. In agreement, we identified a *de novo* *KRAS*^*G13D*^ in myeloid *KMT2A-MLLT3-*expressing cells that had expanded upon secondary transplantation. Altogether, this shows that constitutively active signaling mutations can substitute for external factors and influence the phenotype of the developing *KMT2A-*R leukemia, at least in xenograft models.

## Accession code

GSE127492.

## Supplementary information

Supplementary Information

Supplementary Data 1

Supplementary Data 2

Supplementary Data 3

Supplementary Data 4

Supplementary Data 5

Supplementary Data 6

Supplementary Data 7

Supplementary Data 8

Supplementary Data 9
