# Supplementary material for: FLT3N676K drives acute myeloid leukemia in a xenograft model of KMT2A-MLLT3 leukemogenesis
Source: Leukemia. 2019 Apr 5;33(9):2310–4. doi: 10.1038/s41375-019-0465-1 (PMC6756218; doi:10.1038/s41375-019-0465-1)
Supplement: Supplementary file 1 — Supplementary Information [file 41375_2019_465_MOESM1_ESM.pdf]

Supplementary Information

***FLT3^{N676K}* drives acute myeloid leukemia in a xenograft model of *KMT2A-MLLT3* leukemogenesis**

Hyrenius-Wittsten et al.

SUPPLEMENTARY MATERIAL AND METHODS

Vectors and virus production

All vectors were based on the Murine Stem Cell Virus (MSCV) backbone and the following vectors were used: MSCV-KMT2A-MLLT3-IRES-mCherry, MSCV-FLT3^{N676K}-IRES-GFP, as well as empty vectors (MSCV-GFP and MSCV-mCherry) lacking transgenes as controls. Retroviral supernatants were produced according to standard protocols using transient transfection of HEK293T cells and viral containing medium was harvested 36h later and stored at -80˚C.

Isolation and retroviral transduction of CD34$^+$ CB cells

Collection and use of human CB was performed after informed and written consent in accordance with the Declaration of Helsinki and was approved by the Lund/Malmö Ethical Committee. Mononuclear cells were isolated through density-gradient centrifugation using Lymphoprep (Axis-Shield, Oslo, Norway) and CD34$^+$ cells were enriched by magnetic-activated cell sorting (Miltenyi Biotech, Bergisch Gladbach, Germany) according to the manufacturer's instructions. Cells were pre-stimulated for 48h in DMEM (Thermo Scientific, Logan, UT, USA) supplemented with 100 units/mL Penicillin and 100 g/ml Streptomycin (Thermo Scientific), 10% fetal bovine serum (Thermo Scientific), 100ng/ml SCF, 50ng/ml FLT3-ligand, and 50ng/ml hTPO (all cytokines were purchased from Peprotech, Rocky Hill, NJ, USA). Retroviral co-transduction was performed as previously described[1].

In vivo xenotransplantation assay

All mice were bred and maintained in accordance with Lund University's ethical regulations and approved by the Swedish Board of Agriculture, Malmö/Lund animal ethics committee in Lund, Sweden. All experiments were performed using 8-12 weeks old male NOD.Cg-

Prkdc^{scid}*Il2rgtm*^{1Wjl}/SzJ (NSG) mice. Mice were conditioned by 200cGy sub-lethal irradiation 18-20h prior to transplantation. Primary recipients were transplanted by tail-vein injection of 0.15-1.00x10^6 unfractionated cells (**Supplementary Data 1**) 16-18h after co-transduction. For secondary transplantations 1x10^6 unfractionated primary cells were transplanted. Mice were kept on Ciprofloxacin (KRKA, Stockholm, Sweden) supplemented drinking water throughout the experiments and monitored daily. When moribund, a peripheral blood (PB) sample was taken from vena saphena after which mice were sacrificed. Remaining bones and spleen were made into single-cell suspension by manual trituration and bone marrow and spleen cells were viably frozen. Complete blood cell count was performed on PB using Micros 60 CS (ABX diagnostics, Montpellier, France).

Flow cytometry and fluorescent-activated cell sorting

Before immunophenotypic analysis, xenograft samples were blocked using 10% mouse serum (Sigma-Aldrich, St. Louis, MO, USA) for 20min at 4˚C. Samples were subsequently stained for 20-30min at 4˚C using the following antibodies: CD45 (clone HI30, #555485, BD), CD3ε (clone HIT3a, #300316, Biolegend, San Diego, CA, USA), CD3ε (clone UCHT1, #300426, Biolegend), CD19 (clone HIB19, #302217, Biolegend), CD33 (clone WM53, #25-0338-42, eBioscience, San Diego, CA, USA), and CD33 (clone WM53, #303416, Biolegend). Dead cells were excluded using Draq7 (Biostatus, Shepshed, United Kingdom). Flow cytometric analysis and fluorescence-activating cell sorting were performed on FACS Aria Fusion (BD, Franklin Lakes, NJ, USA). Data analysis was performed using the FlowJo software (FlowJo, LLC, Ashland, OR, USA).

DNA extraction, amplicon sequencing and analysis of DNA sequencing data

DNA was extracted from flow sorted $hCD45^+$, $hCD45^+CD33^+$, $hCD45^+CD19^+$, and/or $hCD45^+CD19^+CD33^+$ BM cells from mouse h11.13, h11.13-1, and h11.13-2 (**Supplementary Table 2** and **Supplementary Data 1,2**) using the QIAamp DNA Micro Kit according to the manufacturer's instructions and DNA quantity was assessed by the Qubit Fluorometer (Life Technologies). Untransduced human $CD34^+$ CB cells used as donor cells for transplantation acted as germline control.

Sequencing was performed at the Center for Translational Genomics, Lund University using the TruSight myeloid sequencing panel (Illumina) which includes exons and hotspot regions of 54 genes (https://www.illumina.com/products/by-type/clinical-research-products/trusight-myeloid.html#gene-list). In addition, specific hotspot regions of *CEBPA* and *FLT3*, were enforced by a separate PCR amplification (*FLT3*; chr13:28607900-26608700, *CEBPA*; chr19:33792100-33793500, Hg19/GRCh37) followed by library preparation (NexteraXT DNA library preparation kit, Illumina). 2 × 150 bp paired-end sequencing was performed with a NextSeq500 instrument (Illumina) and reads were aligned to hg19 using BWA (0.7.15)[2]. Variant calling was achieved using FreeBayes (1.0.2)[1,3], MuTect (3.7)[2,4] and pindel (0.2.5b9)[5] with a corresponding germline control as reference material to utilize somatic variant calling. A coverage of ≥100X and a variant allele frequency (VAF) >5% was applied as detection limit in the analysis.

Validation of somatic mutations

Validation of mutations was performed in matched primary, secondary recipient, and constitutional DNA, using PCR amplicon deep sequencing. PCR was used to amplify the region covering $KRAS^{G13D}$ using the Platinum Taq DNA Polymerase kit (Life Technologies) according to the manufacturer's instructions and products were analyzed using gel electrophoresis (primer sequences are available upon request). PCR amplicons were purified using AMPure XP beads

(Beckman Coulter Inc., Brea, CA) and prepared for sequencing using the Nextera XT DNA Sample Preparation Kit and Index Kit (Illumina). 2 × 150 bp paired-end sequencing was performed using the Illumina NextSeq 500 (Illumina). Adapters were trimmed using Trimmomatic (0.32) and the trimmed paired-end reads were aligned to hg19 using BWA (0.7.15)[2]. Variant calling was performed using samtools mpileup (1.3.1)[6] and varscan (2.4.1)[7].

RNA extraction, RNA sequencing, and analysis of RNA sequencing data

RNA extraction and library preparation was performed as previously described[1] and 2x80bp paired-end sequencing was performed on Illumina NextSeq 500 (Illumina). Paired-end reads from RNA sequencing were aligned to the human genome hg19 using STAR[8]. Transcript expression levels were estimated as fragments per kilobase of transcript per million mapped fragments (FPKM) and gene FPKM was calculated and normalized using Cufflink[9]. An FPKM cutoff of ≥0.5 was used to define "expressed" genes and data was log2 transformed. When expression data was combined with data sets generated elsewhere[10,11], quantile normalization was performed using R (3.4.2) (RStudio, Inc., Boston, MA, USA) and the limma package (3.34.9)[12]. Visualization and statistical analysis of the RNA sequencing data was performed using Qlucore Omics Explorer 3.4 (Qlucore, Lund, Sweden). Gene set enrichment analyses were performed using GSEA v2.2.0[13,14] with pre-ranked gene lists.

Statistical analysis

Differences between groups were assessed by Mann-Whitney U test. Correlation was assessed using Spearman's rank correlation coefficient. Statistical analysis of survival curves was performed using Mantel-Cox log-rank test. All graphs show mean with all individual data points. All analyses were performed with Prism software version 7.0a (GraphPad software).

SUPPLEMENTARY FIGURES AND TABLES

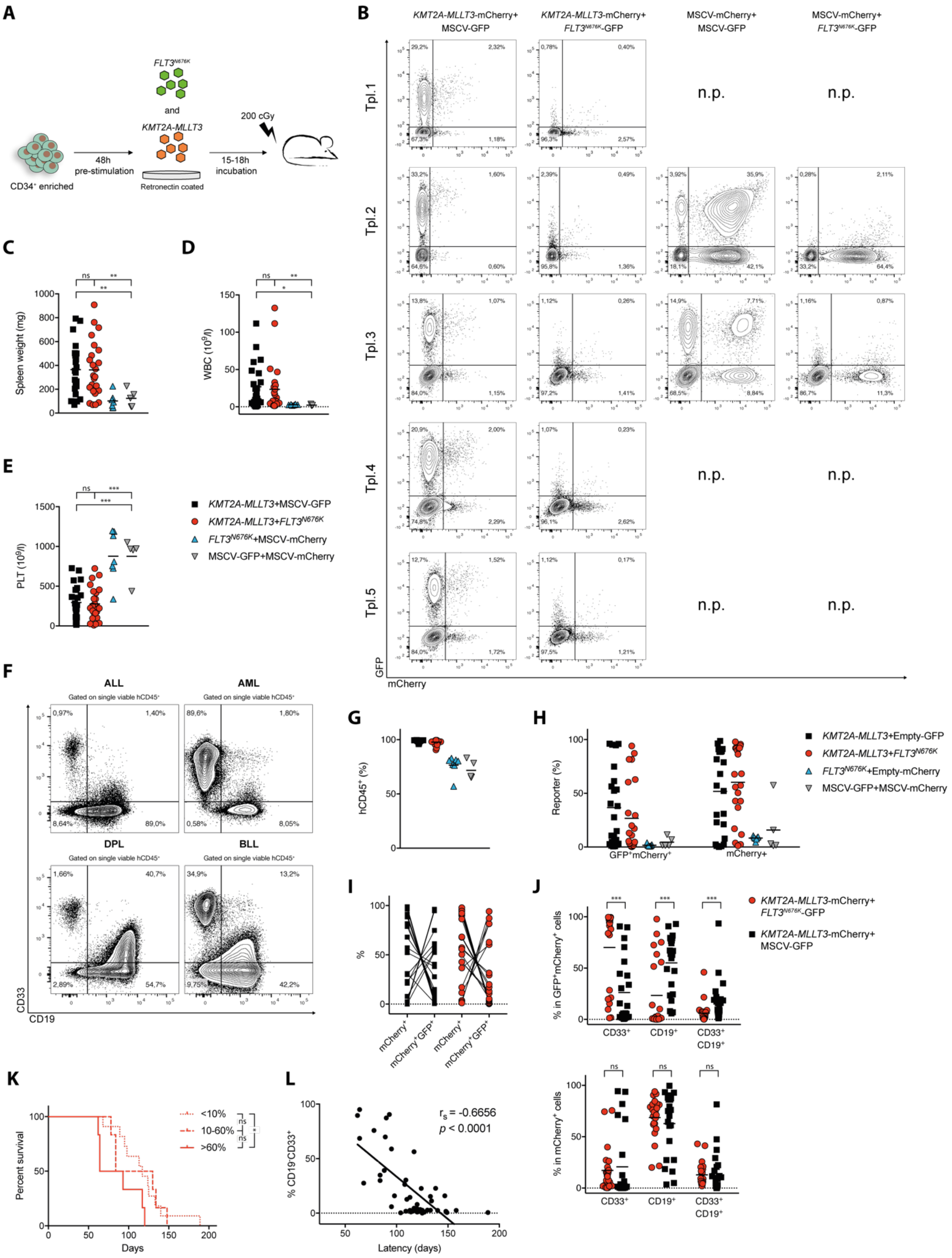

Supplementary Figure 1. (**A**) Schematic experimental outline of the retroviral xenograft transplantation assay. (**B**) Transduction efficiencies 48 hours post-transduction for all

transplantations conducted. (**C**) Spleen weight of sacrificed mice. (**D**) White blood cell count (WBC) of sacrificed mice. (**E**) Platelet count in the peripheral blood of sacrificed mice. (**F**) Flow cytometric analysis revealed the presence of myeloid-, lymphoid-, or double-positive cells within human CD45$^+$ (hCD45$^+$) BM cells in diseased *KMT2A-MLLT3* mice with or without *FLT3^{N676K}*. The disease was defined as acute lymphoblastic leukemia (ALL, >50% CD19$^+$CD33$^-$ cells), acute myeloid leukemia (AML, >50% CD19$^-$CD33$^+$ cells), double-positive leukemia (DPL, >20% CD19$^+$CD33$^+$ cells), or bilineal leukemia (BLL, <50% CD19$^+$CD33$^-$, <50% CD19$^-$CD33$^+$, and <20% CD19$^+$CD33$^+$ cells). (**G**) Distribution of human CD45$^+$ (hCD45$^+$) cells in the BM of transplanted mice. (**H**) Distribution of GFP and mCherry expressing cells in hCD45$^+$ BM cells. (**I**) Paired distribution of mCherry$^+$ and mCherry$^+$GFP$^+$ cells within hCD45$^+$ BM cells for *KMT2A-MLLT3+* MSCV-GFP and *KMT2A-MLLT3+FLT3^{N676K}* recipients. (**J**) Mature lineage distribution in *FLT3^{N676K}*-GFP$^+$+*KMT2A-MLLT3*-mCherry$^+$ and MSCV-GFP$^+$+*KMT2A-MLLT3*-mCherry$^+$ BM cells using CD33 and CD19 showed a significantly higher fraction of CD33$^+$ myeloid cells in *KMT2A-MLLT3* cells that co-expressed *FLT3^{N676K}*. (**K**) Kaplan-Meier survival curves of NSG mice transplanted with CD34$^+$ cord blood cells co-transduced with *KMT2A-MLLT3+FLT3^{N676K}* divided on fraction of mCherry$^+$GFP$^+$, <10% (n=11), 10-60% (n=6), or >60% (n=6), cells within hCD45$^+$ BM cells, Mantel-Cox log-rank test. (**L**) Spearman's correlation showed a significant correlation between the fraction of CD19$^-$CD33$^+$ myeloid cells in BM at the time of sacrifice and disease latency (r_s=-0.6537, P<0.0001). Mann-Whitney U test used in C-E, J. * $P \leq 0.05$, ** $P \leq 0.01$, *** $P \leq 0.001$, n.p. = not performed, ns = not significant.

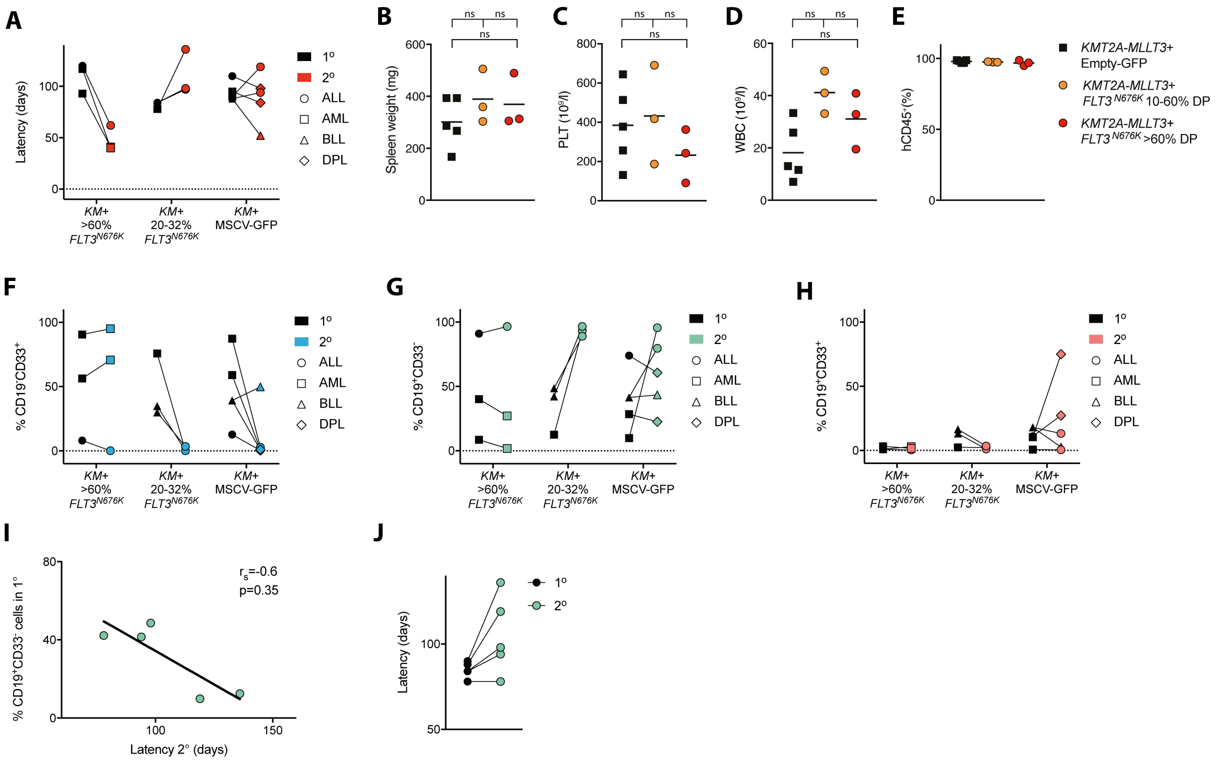

Supplementary Figure 2. (**A**) Paired disease latency for primary- and secondary *KMT2A-MLLT3* leukemias with >60% $FLT3^{N676K}$ (*KM*+>60% $FLT3^{N676K}$), 20-32% $FLT3^{N676K}$ (*KM*+20-32% $FLT3^{N676K}$), or MSCV-GFP (*KM*+MSCV-GFP). (**B**) Spleen weight- (**C**) platelet count in the peripheral blood, and (**D**) white blood cell count (WBC) of sacrificed secondary mice. (**E**) Distribution of human $CD45^+$ ($hCD45^+$) cells in the BM of transplanted secondary mice. (**F**) Progression of $CD19^-CD33^+$ myeloid *KMT2A-MLLT3* leukemia cells with >60% $FLT3^{N676K}$ (*KM*+>60% $FLT3^{N676K}$), 20-32% $FLT3^{N676K}$ (*KM*+20-32% $FLT3^{N676K}$), or MSCV-GFP (*KM*+MSCV-GFP) in secondary recipients. (**G**) Progression of $CD19^+CD33^-$ lymphoid *KMT2A-MLLT3* leukemia cells with *KM*+>60% $FLT3^{N676K}$, *KM*+20-32% $FLT3^{N676K}$, or *KM*+MSCV-GFP in secondary recipients. (**H**) Progression of $CD19^+CD33^+$ double-positive *KM*+>60% $FLT3^{N676K}$, *KM*+10-60% $FLT3^{N676K}$, or *KM*+MSCV-GFP in secondary recipients. (**I**) Spearman´s rank correlation between frequency of $CD19^+CD33^-$ leukemia cells in the primary BM and the resultant disease latency of secondary recipients. (**J**) Paired disease latency in primary- and secondary *KMT2A-MLLT3*+MSCV-GFP ALLs. Statistical testing on paired

data (A, F-H, J) was not performed due to the small sample size; Mann-Whitney U test used in B-D. ns = not significant.

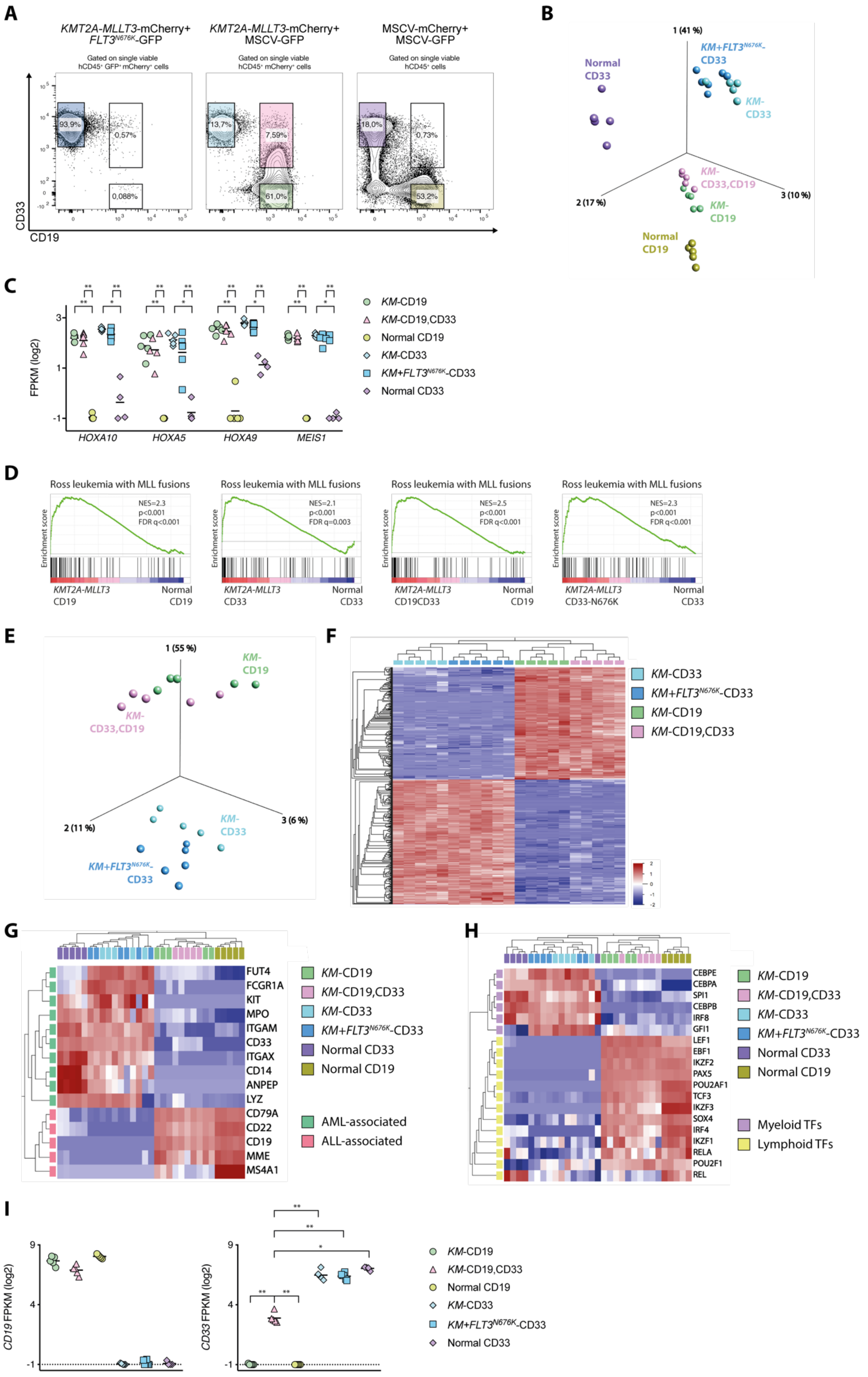
A
KMT2A-MLLT3-mCherry+ FLT3N676K-GFP
Gated on single viable hCD45+ GFP+ mCherry+ cells
93,9%
0,57%
0,088%
KMT2A-MLLT3-mCherry+ MSCV-GFP
Gated on single viable hCD45+ mCherry+ cells
13,7%
7,59%
61,0%
MSCV-mCherry+ MSCV-GFP
Gated on single viable hCD45+ cells
18,0%
0,73%
53,2%
CD33
CD19
B
1 (41 %)
2 (17 %)
3 (10 %)
Normal CD33
KM+FLT3N676K-CD33
KM-CD33
KM-CD33,CD19
KM-CD19
Normal CD19
C
FPKM (log2)
HOXA10
HOXA5
HOXA9
MEIS1
KM-CD19
KM-CD19,CD33
Normal CD19
KM-CD33
KM+FLT3N676K-CD33
Normal CD33
D
Ross leukemia with MLL fusions
Enrichment score
NES=2.3 p<0.001 FDR q<0.001
KMT2A-MLLT3 CD19
Normal CD19
NES=2.1 p<0.001 FDR q=0.003
KMT2A-MLLT3 CD33
Normal CD33
NES=2.5 p<0.001 FDR q<0.001
KMT2A-MLLT3 CD19CD33
Normal CD19
NES=2.3 p<0.001 FDR q<0.001
KMT2A-MLLT3 CD33-N676K
Normal CD33
E
1 (55 %)
2 (11 %)
3 (6 %)
KM-CD19
KM-CD33,CD19
KM-CD33
KM+FLT3N676K-CD33
F
KM-CD33
KM+FLT3N676K-CD33
KM-CD19
KM-CD19,CD33
G
FUT4
FCGR1A
KIT
MPO
ITGAM
CD33
ITGAX
CD14
ANPEP
LYZ
CD79A
CD22
CD19
MME
MS4A1
KM-CD19
KM-CD19,CD33
KM-CD33
KM+FLT3N676K-CD33
Normal CD33
Normal CD19
AML-associated
ALL-associated
H
CEBPE
CEBPA
SPI1
CEBPB
IRF8
GFI1
LEF1
EBF1
IKZF2
PAX5
POU2AF1
TCF3
IKZF3
SOX4
IRF4
IKZF1
RELA
POU2F1
REL
KM-CD19
KM-CD19,CD33
KM-CD33
KM+FLT3N676K-CD33
Normal CD33
Normal CD19
Myeloid TFs
Lymphoid TFs
I
CD19 FPKM (log2)
CD33 FPKM (log2)
KM-CD19
KM-CD19,CD33
Normal CD19
KM-CD33
KM+FLT3N676K-CD33
Normal CD33

Supplementary Figure 3. (**A**) Gating strategy for leukemia populations sorted for RNA sequencing, with MSCV-GFP+MSCV-mCherry samples gated on single viable hCD45$^+$ cells, *KMT2A-MLLT3*+MSCV-GFP gated on single viable hCD45$^+$mCherry$^+$ cells, and *KMT2A-MLLT3+FLT3*^{N676K} gated on single viable hCD45$^+$mCherry$^+$GFP$^+$ cells. (**B**) Unsupervised (10019 variables) principal component analysis (PCA) of myeloid CD19$^-$CD33$^+$ leukemia cells from *KMT2A-MLLT3*+MSCV-GFP (*KM*-CD33) and *KMT2A-MLLT3+FLT3*^{N676K} (*KM+FLT3*^{N676K}-CD33), lymphoid CD19$^+$CD33$^-$ leukemia cells from *KMT2A-MLLT3*+MSCV-GFP (*KM*-CD19), and double-positive CD19$^+$CD33$^+$ leukemia cells from *KMT2A-MLLT3*+MSCV-GFP (*KM*-CD19,CD33), as well as normal myeloid- CD19$^-$CD33$^+$ (Normal CD33) and lymphoid CD19$^+$CD33$^-$ (Normal CD19) cells from MSCV-GFP+MSCV-mCherry. (**C**) Expression (FPKM log2) of the *KMT2A-MLLT3* associated genes *HOXA10, HOXA5, HOXA9,* and *MEIS1* within the sorted leukemia populations. (**D**) GSEA revealed that pediatric leukemia *KMT2A*-signatures were enriched in *KMT2A-MLLT3* and *KMT2A-MLLT3+FLT3*^{N676K} leukemia irrespective of lineage. (**E**) Unsupervised (8638 variables) principal component analysis of highly purified leukemia cells from myeloid CD19$^-$CD33$^+$ *KMT2A-MLLT3*+MSCV-GFP (*KM*-CD33) and *KMT2A-MLLT3+FLT3*^{N676K} (*KM+FLT3*^{N676K}-CD33), lymphoid CD19$^+$CD33$^-$ *KMT2A-MLLT3*+MSCV-GFP (*KM*-CD19), and double-positive CD19$^+$CD33$^+$ *KMT2A-MLLT3*+MSCV-GFP (*KM*-CD19,CD33). (**F**) Hierarchical clustering based on multigroup comparison of *KM*-CD33, *KM+FLT3*^{N676K}-CD33, *KM*-CD19, and *KM*-CD19,CD33 using 501 variables (P=2.8e^{-11}, FDR=4.8e^{-10}). (**G**) Hierarchical clustering of leukemia samples based on markers clinically associated with ALL and AML[17]. (**H**) Hierarchical clustering of leukemia samples based on lymphoid- and myeloid transcription factors[15,16] (19 variables). (**I**) Expression (FPKM log2) of the surface markers *CD19* and *CD33* within the purified populations. Mann-Whitney U test used in C and I, * $P\leq$0.05, * $P\leq$0.01.

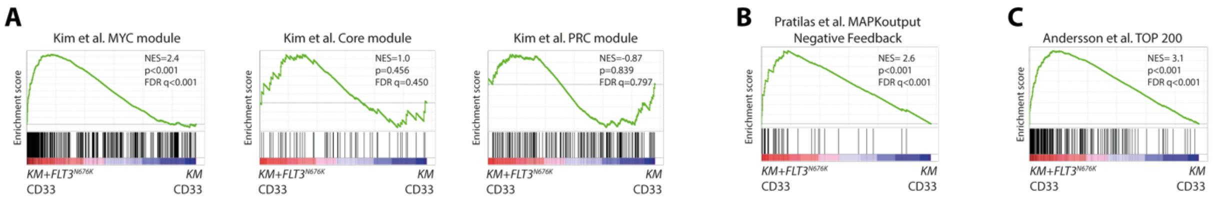

Supplementary Figure 4. (**A**) GSEA revealed enrichment of a described MYC module[11,18] (but not Core- or polycomb (PRC) modules). GSEA revealed enrichment of a described a (**B**) MEK/ERK output and negative feedback signature[19] for AML *KMT2A-MLLT3* leukemia samples with *FLT3*^{N676K}, and (**C**) a signature of the top 200 genes that discriminate infant *KMT2A-AFF1* B-ALL patients harboring activating mutations to those lacking such mutations[20].

Supplementary Table 1. TruSight myeloid sequencing panel coverage data.

Total reads	Mapped Reads	Average Coverage	% >100X	% >500X
8683323	8621887	10031	98.53	97.55

Supplementary Table 2. Identified *de novo* mutations from the targeted- and validation sequencing, their variant allele frequency (VAF).

Subject ID	Population	Gene symbol	Reference transcript	Position	Ref	Alt	AA Change	VAF (TruSight)	Coverage (TruSight)	VAF (validation)	Validation Alt. Reads	Validation Tot. Reads
h11.13	hCD45+	*KRAS*	NM_033360.2	Chr12:25398281	G	T	G13D	n.a.	n.a.	0	0	693
h11.13	hCD45+CD33+	*KRAS*	NM_033360.2	Chr12:25398281	G	T	G13D	n.a.	n.a.	0	0	709
h11.13	hCD45+CD19+	*KRAS*	NM_033360.2	Chr12:25398281	G	T	G13D	n.a.	n.a.	0	0	726
h11.13-1	hCD45+	*KRAS*	NM_033360.2	Chr12:25398281	G	T	G13D	0.18	15100x	0.17	169	894
h11.13-1	hCD45+CD33+	*KRAS*	NM_033360.2	Chr12:25398281	G	T	G13D	n.a.	n.a.	0.51	603	1179
h11.13-1	hCD45+CD19+	*KRAS*	NM_033360.2	Chr12:25398281	G	T	G13D	n.a.	n.a.	0	0	698
h11.13-2	hCD45+	*KRAS*	NM_033360.2	Chr12:25398281	G	T	G13D	n.a.	n.a.	0	0	829
h11.13-2	hCD45+CD19+	*KRAS*	NM_033360.2	Chr12:25398281	G	T	G13D	n.a.	n.a.	0	0	630
h11.13-2	hCD45+CD19+CD33+	*KRAS*	NM_033360.2	Chr12:25398281	G	T	G13D	n.a.	n.a.	0	0	698
Donor	hCD45+CD34+	*KRAS*	NM_033360.2	Chr12:25398281	G	T	G13D	n.a.	n.a.	0	0	730

n.a. = not applicable

Supplementary Table 3. Sorting strategy for leukemic and normal populations.

**Sorted normal population**	**Gating strategy (pre-gated on live single hCD45^{+} cells)**
KM-ALL	mCherry^{+}, CD19^{+}, CD33^{-}
KM-DPL	mCherry^{+}, CD19^{+}, CD33^{+}
KM-AML	mCherry^{+}, CD19^{-}, CD33^{+}
KM+FLT3^{N676K}-AML	mCherry^{+}, GFP^{+}, CD19^{-}, CD33^{+}
Normal lymphoid	CD19^{+}, CD33^{-}
Normal myleoid	CD19^{-}, CD33^{+}

SUPPLEMENTARY REFERENCES

1 Hyrenius-Wittsten A, Pilheden M, Sturesson H, Hansson J, Walsh MP, Song G *et al.* De novo activating mutations drive clonal evolution and enhance clonal fitness in KMT2A-rearranged leukemia. *Nat Commun* 2018; **9**: 1770.

2 Li H, Durbin R. Fast and accurate short read alignment with Burrows-Wheeler transform. *Bioinformatics* 2009; **25**: 1754–1760.

3 *https://arxiv.org/abs/1207.3907*.

4 Cibulskis K, Lawrence MS, Carter SL, Sivachenko A, Jaffe D, Sougnez C *et al.* Sensitive detection of somatic point mutations in impure and heterogeneous cancer samples. *Nat Biotechnol* 2013; **31**: 213–219.

5 Ye K, Schulz MH, Long Q, Apweiler R, Ning Z. Pindel: a pattern growth approach to detect break points of large deletions and medium sized insertions from paired-end short reads. *Bioinformatics* 2009; **25**: 2865–2871.

6 Li H, Handsaker B, Wysoker A, Fennell T, Ruan J, Homer N *et al.* The Sequence Alignment/Map format and SAMtools. *Bioinformatics* 2009; **25**: 2078–2079.

7 Koboldt DC, Zhang Q, Larson DE, Shen D, McLellan MD, Lin L *et al.* VarScan 2: somatic mutation and copy number alteration discovery in cancer by exome sequencing. *Genome Res* 2012; **22**: 568–576.

8 Dobin A, Davis CA, Schlesinger F, Drenkow J, Zaleski C, Jha S *et al.* STAR: ultrafast universal RNA-seq aligner. *Bioinformatics* 2013; **29**: 15–21.

9 Trapnell C, Williams BA, Pertea G, Mortazavi A, Kwan G, van Baren MJ *et al.* Transcript assembly and quantification by RNA-Seq reveals unannotated transcripts and isoform switching during cell differentiation. *Nat Biotechnol* 2010; **28**: 511–515.

10 Lilljebjörn H, Henningsson R, Hyrenius-Wittsten A, Olsson L, Orsmark-Pietras C, Palffy von S *et al.* Identification of ETV6-RUNX1-like and DUX4-rearranged subtypes in paediatric B-cell precursor acute lymphoblastic leukaemia. *Nat Commun* 2016; **7**: 11790.

11 Corces MR, Buenrostro JD, Wu B, Greenside PG, Chan SM, Koenig JL *et al.* Lineage-specific and single-cell chromatin accessibility charts human hematopoiesis and leukemia evolution. *Nat Genet* 2016; **48**: 1193–1203.

12 Ritchie ME, Phipson B, Wu D, Hu Y, Law CW, Shi W *et al.* limma powers differential expression analyses for RNA-sequencing and microarray studies. *Nucleic Acids Res* 2015; **43**: e47.

13 Mootha VK, Lindgren CM, Eriksson K-F, Subramanian A, Sihag S, Lehar J *et al.* PGC-1alpha-responsive genes involved in oxidative phosphorylation are coordinately downregulated in human diabetes. *Nat Genet* 2003; **34**: 267–273.

14 Subramanian A, Tamayo P, Mootha VK, Mukherjee S, Ebert BL, Gillette MA *et al.* Gene set enrichment analysis: a knowledge-based approach for interpreting genome-wide expression profiles. *Proc Natl Acad Sci USA* 2005; **102**: 15545–15550.

15 Rosenbauer F, Tenen DG. Transcription factors in myeloid development: balancing differentiation with transformation. *Nat Rev Immunol* 2007; **7**: 105–117.

16 Matthias P, Rolink AG. Transcriptional networks in developing and mature B cells. *Nat Rev Immunol* 2005; **5**: 497–508.

17 Peters JM, Ansari MQ. Multiparameter flow cytometry in the diagnosis and management of acute leukemia. *Arch Pathol Lab Med* 2011; **135**: 44–54.

18 Kim J, Woo AJ, Chu J, Snow JW, Fujiwara Y, Kim CG *et al.* A Myc network accounts for similarities between embryonic stem and cancer cell transcription programs. *Cell* 2010; **143**: 313–324.

19 Pratilas CA, Taylor BS, Ye Q, Viale A, Sander C, Solit DB *et al.* (V600E)BRAF is associated with disabled feedback inhibition of RAF-MEK signaling and elevated transcriptional output of the pathway. *Proc Natl Acad Sci USA* 2009; **106**: 4519–4524.

20 Andersson AK, Ma J, Wang J, Chen X, Gedman AL, Dang J *et al.* The landscape of somatic mutations in infant MLL-rearranged acute lymphoblastic leukemias. *Nat Genet* 2015; **47**: 330–337.
